# Adverse renal effects associated with cardiopulmonary bypass

**DOI:** 10.1177/02676591231157055

**Published:** 2023-02-16

**Authors:** Benjamin Milne, Tom Gilbey, Filip De Somer, Gudrun Kunst

**Affiliations:** 1 Department of Anaesthesia & Pain Medicine, 8948 King’s College Hospital NHS Foundation Trust, London, UK; 2 Nuffield Department of Anaesthesia, John Radcliffe Hospital, Oxford, UK; 3 Department of Human Structure and Repair, Faculty of Medicine and Health Sciences, 60200 Ghent University Hospital, Ghent, Belgium; 4 School of Cardiovascular and Metabolic Medicine and Sciences, 406774 King’s College London British Heart Foundation Centre of Excellence, London, UK

**Keywords:** cardiac, surgery, bypass, kidney, injury

## Abstract

Cardiac surgery on cardiopulmonary bypass (CPB) is associated with postoperative renal dysfunction, one of the most common complications of this surgical cohort. Acute kidney injury (AKI) is associated with increased short-term morbidity and mortality and has been the focus of much research. There is increasing recognition of the role of AKI as the key pathophysiological state leading to the disease entities acute and chronic kidney disease (AKD and CKD). In this narrative review, we will consider the epidemiology of renal dysfunction after cardiac surgery on CPB and the clinical manifestations across the spectrum of disease. We will discuss the transition between different states of injury and dysfunction, and, importantly, the relevance to clinicians. The specific facets of kidney injury on extracorporeal circulation will be described and the current evidence evaluated for the use of perfusion-based techniques to reduce the incidence and mitigate the complications of renal dysfunction after cardiac surgery.

## Introduction

Cardiac surgery is associated with a spectrum of adverse renal events, including acute kidney injury (AKI), acute kidney disease (AKD) and chronic kidney disease (CKD). The AKI state is associated with increased short-term morbidity and mortality, before potentially transitioning into AKD and CKD, with their attendant effect on longer-term outcomes.^1,2^ As the key to both early and late disease burden, there has been much interest in the study of cardiac surgery-associated AKI (CSA-AKI).

CSA-AKI is best defined by Kidney Disease Improving Global Outcomes (KDIGO) criteria as an AKI within one-week of cardiac surgery.^3,4^ It is the outcome of numerous pathophysiological insults, one of which is the use of extracorporeal oxygenation and perfusion using cardiopulmonary bypass (CPB). Despite this major role in the pathogenesis of CSA-AKI, the avoidance of CPB has not reliably reduced the incidence of CSA-AKI or longer-term renal outcomes.^5–8^ This evidence does not diminish the importance of CPB in causing renal dysfunction, but rather reflects the importance of the multifactorial aetiology of the disease, and in particular the importance of the haemodynamic changes during off-pump surgery.^9^

This narrative review will describe the specific pathophysiology of CPB use with respect to renal dysfunction, and the impact it has upon the three domains of renal dysfunction (i.e. AKI, AKD and CKD) will be discussed. Finally, an updated state of the literature with relation to perfusion-based strategies will be considered.

## Definitions and epidemiology

### Acute kidney injury

CSA-AKI is a state of potentially reversible acute organ damage or dysfunction defined by deranged functional markers (serum creatinine (sCr) or urine output (UO), most appropriately using the thresholds sets out in the KDIGO criteria) within one week of cardiac surgery.^3,10^ This term incorporates the entity of AKI-CPB (AKI occurring after CPB), a major contributor to CSA-AKI, however it is a more specific term with overlapping pathophysiology.

The incidence of CSA-AKI is in the range of 5–40%.^3,11^ The incidence of lower severity injury is greater than for more severe injury, although the idiosyncrasies of cardiac surgery often render the staging criteria less accurate than in other contexts.^12,13^ A more specific incidence of AKI-CPB is 20–30%, with up to 5% requiring renal replacement therapy (RRT).^14,15^ AKI-CPB is associated with a two-fold increase in early mortality regardless of the AKI definition employed.^16^

**Figure 1.**
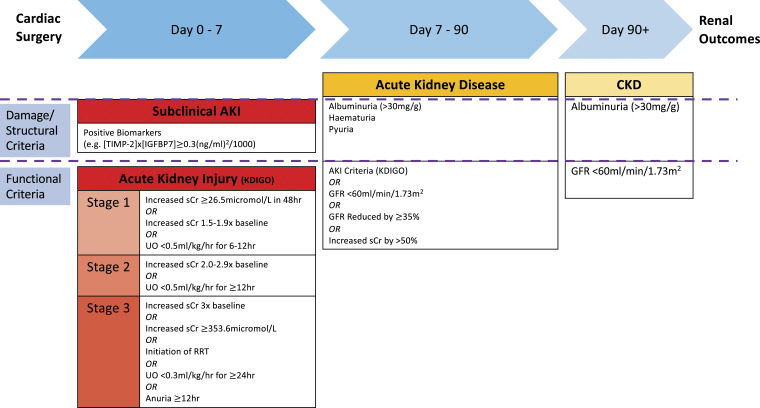
Diagnosing renal dysfunction following cardiac surgery. AKI: Acute kidney injury; CKD: Chronic kidney disease; GFR: Glomerular filtration rate; IGFBP7: Insulin growth factor binding protein 7; KDIGO: Kidney disease improving glocal outcomes; RRT: Renal replacement therapy; sCr: Serum creatinine; TIMP-2: Tissue inhibitor of metalloproteinase-2; UO: Urine output.

The incidence of AKI varies by classification system used, with varying sensitivity and specificity amongst the different definitions. One meta-analysis demonstrated a CSA-AKI incidence of 24.2% by KDIGO criteria, compared with 18.9% by RIFLE (Risk Injury Failure Loss End-Stage) and 28.0% by AKIN (Acute Kidney Injury Network) classifications.^12^ The AKIN classification has increased sensitivity for AKI compared to the RIFLE classification, reflecting that small increments in sCr (0.3–0.5 mg/dL) are independently associated with increased 30 days mortality. However, the AKIN classification may miss AKI occurring after 48 hours.^12,17–20^ The KDIGO Criteria combines these two classifications and has further improved sensitivity and predicts in-hospital mortality, although mortality is similar across all three systems.^12,17,21,22^ This classification schema has reached consensus recommendation for use in cardiac surgery (Figure 1).^23^

The KDIGO classification is particularly suited for AKI-CPB as the incorporation of both sCr and UO criteria reflects the unique physiological challenges posed by CPB. Haemodilution from the circuit priming volume results in a postoperative sCr that may be below preoperative baseline and fails to reflect reduced renal function, particularly in the early postoperative period.^23^ Alternatively, in this volume-loaded state UO may be falsely reassuring, however a reduction in this value, irrespective of sCr concentration, is strongly indicative of dysfunction.

Importantly, this classification system is not designed specifically for use in the cardiac surgical population. As such, sCr and UO must be interpreted in the context of current fluid balance (FB) to avoid underdiagnosing AKI after CPB, although the practicalities of this limit clinical applicability.^24^ In a single-centre study (*n* = 1016) early relative changes in sCr adjusted for cumulative FB, were predictive for subsequent AKI after valve surgery.^25^ Furthermore, in another large retrospective single-centre study, adjusted sCr for FB did not reassign any patient from AKI status to non-AKI, but did increase the incidence of AKI (25.3% vs 37.2%, *p* < 0.001). FB-adjusted sCr-diagnosed AKI increased incidence of poor outcomes (including intensive care unit (ICU) mortality and RRT requirement) compared with non-AKI patients. Outcomes were worse in patients with an AKI diagnosis by unadjusted criteria, reflecting that adjustment may increase sensitivity, but the original criteria-based diagnosis is more specific for a more injured state.^26^

Classification using these functional markers (sCr and UO) is further limited by sCr being affected by factors not related to glomerular filtration rate (GFR), including reduced creatinine production in the physiologically deconditioned cardiac surgery patient. Furthermore, these markers do not localise the injury.^21^ Novel biomarkers are of increasing interest and are discussed below.

### Subclinical cardiac surgery-associated-acute kidney injury

Subclinical AKI is a state of renal cellular stress or damage, defined by the presence of raised appropriate biomarkers, in the absence of deranged functional markers such as creatinine.^27^ This entity is of increasing clinical importance as recognition enables earlier intervention, which has heralded improved outcomes in several studies.^28–32^

Novel biomarkers of renal stress or damage have been incorporated into a schema for the identification of a subclinical injury state, which avoids a number of the aforementioned shortcomings of solely using functional markers.^27^ Alternatively, these biomarkers can be used for the prediction of subsequent KDIGO-defined AKI. Examples of biomarkers and their uses during and after CPB are shown in Table 1.^10,27–29,33–41^

**Table 1.** Novel renal cellular stress/damage biomarkers and their potential use in cardiac surgery on CPB.

Novel biomarker	Biomarker origin and function
[TIMP-2]x[IGFBP7]	Cell cycle arrest proteins released after ischaemia-reperfusion injury
	May predict AKI as early as 1 h after commencement of CPB
	Peak intraoperatively and at 6 h postoperatively
	Measurement at 4 h post-CPB has been used to guide a care bundle intervention to reduce incidence of severe CSA-AKI
Neutrophil gelatinase-associated lipocalin (NGAL)	Released by proximal tubular epithelia after injury
	Urinary and serum levels have diagnostic and prognostic functions
	Urinary levels can guide diagnosis from 2 h after CPB and on ICU admission
Cystatin C	Protease inhibitor which is freely filtered by glomerulus
	Crucially, not a stress/damage marker, but a functional marker
	Diagnostic for AKI (serum or urinary)
Kidney injury molecule-1 (KIM-1)	Released into urine after tubular damage
	Prediction of AKI 2 h after CPB
Interleukins (IL) – 6 and 18	Proinflammatory cytokines found in serum after tubular damage
	Prediction of AKI within 6 h after CPB
Free haemoglobin	A trigger of oxidative stress involved in AKI
	Of particular relevance to AKI-CPB, given the risk of haemolysis in the extracorporeal circulation
	An elevated level (compared to baseline) at the end of CPB may predict AKI development

AKI: Acute kidney injury; AKI-CPB: Acute kidney injury after cardiopulmonary bypass; CPB: Cardiopulmonary bypass; CSA-AKI: Cardiac surgery-associated acute kidney injury; ICU: Intensive care unit; IGFBP7: Insulin growth factor binding protein 7; TIMP-2: Tissue inhibitor of metalloproteinase-2.

### Acute kidney disease

AKD has been defined as a condition of KDIGO-defined AKI persisting for ≥ 7 days after the inciting AKI event, in this specific case, cardiac surgery on CPB.^42^ However, this limited definition fails to capture with sufficient sensitivity the spectrum of deterioration in renal function which may occur. As this state represents a key transition phase for permanent impairment, a wider definition with both functional and structural/damage criteria has been proposed (Figure 1). This includes the KDIGO AKI criteria, as well as defined reductions in GFR (GFR <60 mL/min/1.73 m^2^ or decrease in GFR by ≥ 35% from baseline), increased sCr >50%, or development of a significant albuminuria.^43^ Persistence of this state beyond 90 days should be reclassified as CKD.^42^

Cardiac surgery patients are vulnerable to pre-, intra- and postoperative physiological insults, and whilst use of CPB intraoperatively may be the major inciting event, ongoing pathological insults postoperatively can contribute to ongoing dysfunction, which may be different to AKI in other contexts. However, AKD remains a useful concept, marking an ongoing period of potential intervention to improve renal, and other, outcomes. In other settings patients may present with AKD, but within the context of cardiac surgery, with close renal monitoring and often lengthier postoperative inpatient admissions than for major non-cardiac surgeries, the transition is more likely to be clinically observed. However, with increasing uptake of enhanced recovery after cardiac surgery (ERACS) patients may be discharged prior to derangement of functional markers (and an identifiable AKI state), and are later instead recognised as AKD/CKD.^44^

Few studies have specifically investigated AKD after cardiac surgery. In one study, AKD was defined as an increased sCr at least 1.5x baseline >7 days after cardiac surgery, which is crucially different to the recently harmonised definition given above, instead using the previous Acute Disease Quality Initiative (ADQI) 16 workgroup definition.^42^ In this study, 11.2% of all patients developed AKD, and there was a 90 days mortality of 19.9% associated with AKD (adjusted OR for mortality of 63.0 [27.9–190.6]). The adjusted OR for 90 days mortality with AKI alone versus AKD was 8.43 [2.87–27.74] and 63.0 [27.9–180.6] respectively.^45^

### Chronic kidney disease

CKD is a condition of deranged kidney function (GFR <60 mL/min/1.73 m^2^) or evidence of kidney damage (e.g. albuminuria), persisting for >3 months^43^ Few studies have examined the incidence of CKD after cardiac surgery on CPB, but one multi-centre study has suggested an incidence of 5.7%, whilst another study has suggested that incidence at 3 years is 26.1%.^46,47^ CKD is subsequently associated with increased mortality and morbidity, particularly cardiovascular disease.^21^ Furthermore, outcomes of subsequent cardiac surgery, should it be required, are worse with preoperative CKD.^2^

Figure 1 delineates a schema for diagnosis of renal dysfunction after cardiac surgery.^4,42,43^

## Transitions between disease states

Much of the research literature has focused upon stratifying, modifying and mitigating risk for CSA-AKI.^9^ This has been driven by the associated short-term morbidity and mortality of AKI, as well as the related transition to CKD, and a post-CPB AKI incidence of 20–30% translates to a CKD incidence of 6–26%, although this latter phenomenon has been less studied and has a much more complex relationship in this comorbid population.^14,46,47^ However, description of the AKD state allows for the recognition of an ongoing degree of dysfunction and potentially a patient requiring intervention to positively impact their longer-term outcomes.

In one multi-centre retrospective study of patients undergoing surgery on CPB, 42.6% of patients achieved the UO definition of CSA-AKI alone, with most sustaining a Stage 2 injury, whilst 6.1% of patients met the sCr criteria alone, with most having a Stage 1 injury. Furthermore, 32.5% of patients met CSA-AKI definition by both UO and sCr criteria. Moving from no AKI, to isolated oliguria, to isolated azotaemia and to deranged sCr/UO there was increasing incidence of major adverse kidney events (MAKE) at 180 days (a composite of death, dialysis and persistent renal dysfunction) (4.5%, 7.6%, 13.5% and 21.8%, respectively). Importantly in this context, the restrictive AKI definition used (only 72 h postoperative) may increase the relevance of CPB to the observed injury state. Furthermore, it confirms that whilst an oliguria post-CPB is indicative of poor outcomes, an elevated sCr concentration, and both derangements, indicate worsening long-term renal outcomes. However, these findings may be limited in generalisability by the very high incidence of CSA-AKI (81.2%) and the restrictive definition in this study.^48^

In one prospective single-centre study, CSA-AKI was associated with a significantly higher rate of CKD at 2 years follow-up (6.8% vs 0.2%, *p* < 0.001).^49^ In a retrospective study, the hazard ratio (HR) for development of CKD increased with increasing stage of CSA-AKI (Stage 1: HR 3.11 [2.62–4.91], Stage 2/3: HR 13.36 [9.22–18.72]), suggesting that more severe injury state results in impaired long-term recovery. Furthermore, at each AKI stage there was an increased risk of CKD development with persistent (>48 h) compared with transient (≤48 h) AKI, with only transient Stage 1 injury not significantly increasing incidence of CKD.^47^ Similarly in a single-centre observational study CSA-AKI was associated with a greater incidence of subsequent CKD at 12 months (25% vs 9%, *p* < 0.001), with AKI duration >3 days an independent risk factor for CKD.^50^ This finding has been further supported by a retrospective study.^51^

A similar transition has been shown for AKD in an observational study, where 38.6% of patients with transient (<72 h) AKI developed AKD, compared with 74.1% of patients with persistent ( ≥ 72 h, but crucially <7 days) AKI. Therefore, of the overall 47.1% of AKI patients developing AKD, the persistent injury state was more likely to result in AKD.^45^ Further retrospective evidence has been found for this finding.^51^

Taken together, these findings suggest that progression towards more long-term renal dysfunction relates to both severity of injury and duration. However, what is unclear is whether the persistent injury state is the marker of a more severe initial injury, an ongoing or secondary insult, or inadequate cellular resuscitation, which would be modifiable. Further investigation is required and this will have a significant impact upon the recognition of the importance of CPB in this process. Furthermore, in the post-CPB patient, for reasons previously described, the ability of functional markers to accurately stage AKI is less reliable, and this will impact upon the use of standard AKI definitions alone to identify risk of poor outcomes.

Overall, factors associated with transition from AKI to AKD require further assessment. AKI severity is likely to be important, but the nature of this has not been as clearly delineated as for CKD.^52^ Baseline estimated GFR has been demonstrated in an observational study to be associated with development of AKD amongst AKI patients, but was poorly predictive of AKD itself.^45^ In a multivariate analysis in the same study, only non-elective surgery significantly increased the odds for AKD (odds ratio (OR): 2.15 [1.61–2.87]). Attempts to mitigate or reduce transition to AKD by clinical interventions should follow the evidence-based practices reviewed elsewhere.^9^ The list of prognostic disease modifiers includes severity of AKI, the stage of pre-existing CKD, the number of injury episodes, alongside the duration, and the development of proteinuria.^42^

AKD represents a longer period where intervention may be more practical to improve outcomes. In one of the few studies examining outcomes after CPB, renal recovery occurred in only 54.8% of patients developing AKD. AKD was independently associated with increasing estimated GFR decline in the subsequent two years (30% decline OR 1.79 [1.30–2.40], 40% decline OR 2.62 [1.81–3.75], and 50% decline OR 3.56 [2.24–5.57]).^45^ AKD superimposed on CKD likely represents a particularly high-risk state for progression of kidney disease.^42^ Various AKD trajectories have been described, where the degree of renal dysfunction measured by functional markers may be better, worse, or equal to the dysfunction in the preceding AKI state, although there may not have been an initial AKI by standard criteria.^42^

Further research is required to understand the trajectory of AKD post-CPB. Expert consensus has suggested that not all the issues which are relevant to AKI are likely to be so important for AKD, with the most important factor being appropriate changes to drug dosing. Of intermediate importance is the discontinuation of nephrotoxins, optimisation of volume status and perfusion pressure, monitoring of sCr, avoiding use of radiocontrast and consideration of invasive diagnostic investigation. In contrast to AKI, functional haemodynamic monitoring, UO monitoring, use of RRT and ICU support are of low importance in preventing progression of AKD.^43^ One of the crucial elements to enable more appropriate treatment in the post-CPB patient is to identify AKD without preceding AKI, which may be more reflective of an injury state in this context, and should be treated as such.

**Figure 2.**
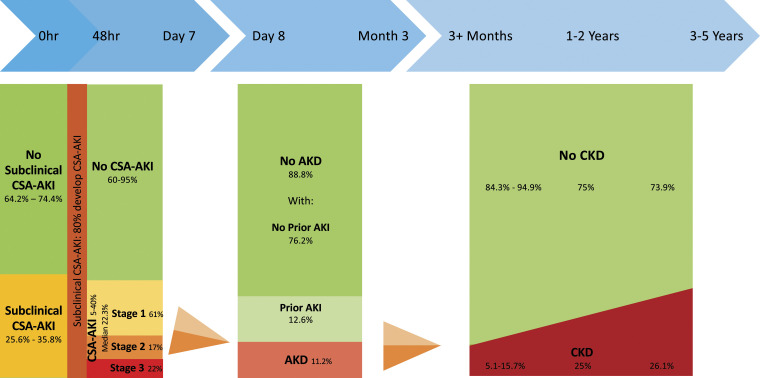
Incidence of renal dysfunction states following the cardiac surgery. AKD: Acute kidney disease; AKI: Acute kidney injury; CSA-AKI: Cardiac surgery-associated acute kidney injury; CKD: Chronic kidney disease. Incidence of subclinical CSA-AKI: Meersch 2017,^28^ Zarbock 2021.^29^ Transition to CSA-AKI: Meersch 2014.^53^ Incidence of AKI: Hu 2016.^12^ Transition to and incidence of AKD: Matsuura 2020.^45^ Transition to CKD: Legouis 2017,^46^ Choe 2021,^47^ Palomba 2017.^50^

Figure 2 shows an evidence-based description of the epidemiology of renal dysfunction after cardiac surgery, illustrating a population approach to disease trajectory.^12,28,29,45–47,50,53^

## Pathophysiology

The pathophysiology of CSA-AKI is multifactorial and the result of numerous synergistic factors across the perioperative period. The spectrum of insults includes microembolisation, neurohormonal activation, nephrotoxins (both endogenous and exogenous), metabolic and haemodynamic factors, inflammation, ischaemia-reperfusion injury, and oxidative stress.

Recent meta-analyses have not unanimously demonstrated a significant difference in CSA-AKI or longer-term renal outcomes between CPB and off-pump procedures, reflecting the impact of the potential haemodynamic insult which still occurs during off-pump surgery.^8,12^ Beyond mere use of CPB, is the consideration of a ‘dose-dependent’ response, with increasing duration of time on CPB and cross-clamp time both reproducibly associated with increased incidence of AKI.^49,54,55^

Alongside perfusion and cross-clamp time, other specific perfusion-related factors in development of AKI-CPB include use of non-pulsatile CPB, haemodilution and hypothermia.^23^ The result of these pathophysiological processes is a kidney with impaired function, persistent renal vasoconstriction, increased sensitivity to exogenous vasoconstrictors, and both vascular endothelial and tubular epithelial cell death.^21^

**Figure 3.**
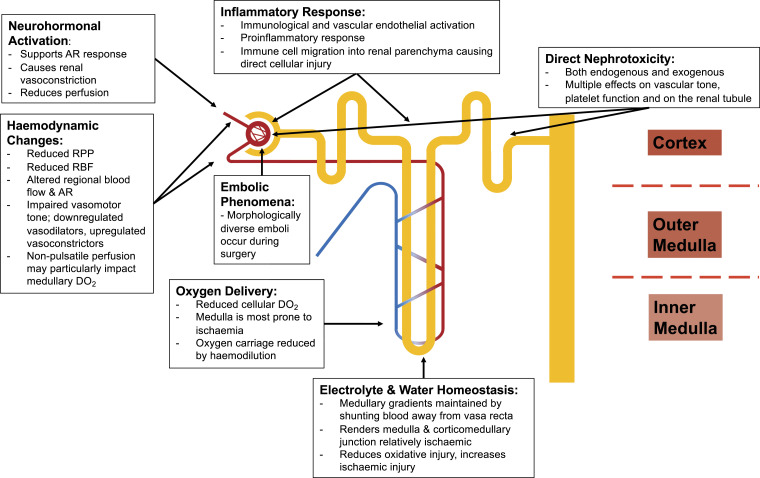
Pathophysiology of renal dysfunction after cardiac surgery on cardiopulmonary bypass. AR: Autoregulation; DO_2_: Oxygen delivery; RBF: Renal blood flow; RPP: Renal perfusion pressure.

The multi-factorial aetiology defies simple classification. An intraoperative (on- or off-pump) or postoperative acute low cardiac output state may cause CSA-AKI (a type 1 cardiorenal syndrome (CRS)). However, description of a type 1 CRS does not account for the multitude of other physiological insults, including the inflammatory effects of surgery and CPB, and the use of potent nephrotoxic and vasoactive medications. Furthermore, the unidirectional relationship this description implies neglects the dynamism of the cardiovascular and renal systems and the contribution of CSA-AKI to postoperative myocardial dysfunction (type 3 CRS) through impaired intravascular fluid handling, metabolic acidaemia, uraemia and hyperkalaemia.^56^ The relevant pathophysiological mechanisms are discussed below, by domain (Figure 3).

### Haemodynamic involvement

The intraoperative haemodynamic changes are superimposed upon the preoperative physiological state, where the patient may have a degree of intravascular volume depletion (secondary to fasting or diuretic use), a high degree of cardiovascular disease, and potential renal or renovascular disease.^15^ At an organism level there may already be a precariously balanced, or failing, systemic circulation, translating to abnormal perfusion at organ level.

Institution of CPB can reduce renal perfusion pressure by 30%, causing regional blood flow and vasomotor tone abnormalities in the kidney.^14^ The organ level autoregulatory response will assist in maintenance of renal blood flow but will occur at a much lower threshold value. A physiological study has clearly delineated the response of regional blood flow on hypothermic CPB, with the kidney particularly affected, with flow reduced by approximately 50% compared with pre-CPB level.^57^ Renal blood flow is also dependent upon the CPB flow rate, but further physiological studies have demonstrated that this is not linear, with maximal organ flow (50 mL/min/100 g tissue) at a CPB flow rate of 2.0 L/min/m^2^, but no increase above this with augmented extracorporeal perfusion.^58^ Renal autoregulatory values have been demonstrated to show good correlation with those for cerebral autoregulation, which may permit near-infrared spectroscopy to demonstrate relevant thresholds.^59^

Abnormal perfusion results in reduced oxygen delivery at a cellular level, exacerbated by extracorporeal haemolysis, which further reduces oxygen carriage causing ischaemia to the renal parenchyma and predisposing to later reperfusion injury.^14^ Oxygen carriage per unit volume of blood will be further reduced by haemodilution within the extracorporeal circuit, although the altered rheology may improve microcirculatory flow should perfusion be adequate.^60^ A nadir oxygen delivery on CPB of <262 mL/min/m^2^ has been associated with development of Stage 2 AKI. As discussed above, increasing pump output may increase end-organ oxygen delivery, but excessive flow may unfavourably increase regional energy consumption resulting in hypoxic stress.^60^ Furthermore, whilst CPB maintains cardiac output, the tissue perfusion pressure is less certain under non-pulsatile conditions.^23^

The autoregulatory response will be impaired by pre- or intraoperative ischaemic injury, and experimental models have demonstrated that there will be a relatively fixed degree of renal vascular resistance, with some remaining vasodilatory ability. The impaired autoregulatory response is a particular concern for further injury during the haemodynamic lability on weaning from CPB.^60^ The likely outcome is a state of relative vasoplegia, and a proinflammatory milieu, with downregulated vasodilators (nitric oxide) and upregulated vasoconstrictors (endothelin, angiotensin-2 and catecholamines), further exacerbating renal injury.^60–62^

Physiological adaptive mechanisms will maintain the electrolyte and water concentration gradients in the renal medulla, which are crucial for homeostatic processes, by shunting the blood filtered in the cortical glomeruli away from the vasa recta. However, this will render the renal medulla and corticomedullary junction relatively hypoxic, protecting against oxidative injury, but increasing the risk of ischaemic injury.^63,64^ The finely balanced cortical and medullary perfusion may also be disturbed by non-pulsatile CPB perfusion, with increased flow to the cortex paradoxically increasing medullary oxygen demand due to the increased solute load, causing corticomedullary ischaemia.^64,65^

Finally, rewarming, including clinically not indicated hyperthermic perfusion, and raised early postoperative temperature, has been associated with an increased risk of AKI, related to the concomitant states of reduced oxygen supply with increasing oxygen demand.^23,66^

### Neurohormonal activation

The neurohormonal response occurs to support the autoregulatory response. A state of sympathetic activation may occur due to preoperative cardiac insult, perioperative cardiac dysfunction and in response to surgical stimulation. The sympathetic hyperactivity, and concomitant activation of the renin-angiotensin-aldosterone system, causes renal vasoconstriction and reduced renal perfusion.^67^

### Inflammatory response

CPB triggers a systemic inflammatory response due to activation of proinflammatory mediators by contact with the extracorporeal membrane of the circuit.^14^ The inflammatory response is further activated by ischaemic-reperfusion injury and oxidative stressors, and these processes lead to immunological and vascular endothelial activation, and the production of further proinflammatory mediators.^60,68,69^ This response involves TNF-alpha, IL-6 and IL-8, the complement system, and reactive oxygen species (ROS) will upregulate proinflammatory transcription factors, such as nuclear factor Kappa B.^14,60,70–72^

The vascular sequelae of the inflammatory response further impairs renal autoregulation. Proinflammatory chemokines and cytokines drive immune cell migration into the renal parenchyma, causing direct cellular injury, manifesting as AKI, and possible longer-term damage, such as fibrosis.^60,71–74^

### Direct nephrotoxicity

Numerous endogenous and exogenous perioperative nephrotoxins have been identified. Of relevance to CPB-AKI is the release of free haemoglobin and its constituents, including iron, due to extracorporeal haemolysis. The release of these nephrotoxins, combined with the depletion or saturation of their endogenous scavengers (transferrin or lactoferrin), can cause altered vascular tone and platelet function, as well as directly injuring the renal tubule.^14,60,75,76^ Free iron catalyses the production of free radicals causing end cellular damage, particularly in the renal epithelium, and worsens oxidative stress upon reperfusion.^60,77^ Free haemoglobin depletes circulating haptoglobin, catalysing free radical production, and forming protein precipitates in the renal collecting system and causing renal arteriole vasoconstriction by reducing nitric oxide concentration. ROS particularly injure the kidney, which normally sequesters free haemoglobin and iron.^64^ Heme-oxygenase-1 is a potential biomarker of interest, being produced by free haemoglobin, and found to be increased in patients with AKI, and associated with increasing CPB duration, haemolysis and inflammation.^78^

### Embolic phenomena

Platelet aggregates, cellular debris, fibrin, fat and air may all embolise during cardiac surgery, and some emboli will be sufficiently small to evade filtration within the extracorporeal circuit.^14^ Fat emboli pose a particular challenge, being readily deformable and thus escape, to a degree, in-line filtration.^79^ This prolonged circulating time can render the endothelial glycocalyx vulnerable to a greater degree of harm.^80^ Atheroembolism can occur during surgical manipulation of the ascending aorta, particularly during cannulation and release of the cross clamp, and likely contribute to CPB-AKI.^60^ Detection of emboli on Doppler studies have been associated with increased risk of postoperative renal dysfunction.^81^

## Proposed perfusion-based approaches to reduce acute kidney injury

### Avoidance of cardiopulmonary bypass

Given the above-described harms of CPB, a potential protective approach would be avoidance of extracorporeal circulation. However, this is clearly not feasible for the performance of anything other than amenable bypass grafts, in the hands of capable surgeons. Furthermore, conflicting evidence has emerged from secondary analyses of two large RCTs for a reduction in CSA-AKI incidence with off-pump versus on-pump surgery, and no clear improvement in longer-term outcomes.^5,7,8^

These findings do not negate the importance of the renal harms of CPB, but instead confirm the presence of other significant perioperative insults and the deleterious effects of the haemodynamic changes of cardiac and major vessel manipulation in off-pump surgery.^9^ Furthermore, whilst duration of CPB has been demonstrated to be associated with incidence of AKI, this is a largely non-modifiable factor.^82^ Therefore, there is a clear requirement for interventions to improve the burden of pathology associated with CPB.

### ‘Miniature’ cardiopulmonary bypass circuits

Miniature (or minimally invasive) extracorporeal circuits (MiECC) enable the use of reduced prime volumes, for example 600 mL compared with 1200 mL.^83^ These have haemocompatible tubing and oxygenator membrane, employ a centrifugal pump and avoid cardiotomy suction and venous reservoir. The theoretical benefits of these circuits are numerous and include a reduction in haemodilution, maintaining a greater haematocrit and reducing blood product transfusion, both of which are reproducibly associated with better renal outcomes after cardiac surgery.^23,84,85^ Furthermore, these alterations allow for the preservation of a greater intravascular volume, and potentially reduce the mechanical haemolysis caused by cardiotomy suction.

A retrospective propensity score matched analysis was performed for patients at a single-centre undergoing CPB on a mini-CPB system (*n* = 104) compared with a conventional circuit (*n* = 601). In this study, there was a representative incidence of AKI (38.8%; using AKIN classification) and RRT requirement (3.8%). The incidence of CSA-AKI for mini-CPB patients was reduced compared with conventional CPB (28.8% vs 40.5%, *p* = 0.03), and in the matched-pair analysis the miniaturised circuit was independently associated with reduced incidence of AKI-CPB (adjusted OR 0.61 [0.38–0.97]). The decision to employ mini-CPB was at the discretion of the surgeon, and although the propensity score matching removed significant differences between the two groups for the final analysis, the small number (*n* = 104 in each group) and single-centre nature limit the wider applicability of these findings.^83^

A subsequent small (*n* = 60) randomised controlled trial (RCT) failed to detect a difference in incidence of AKI (by AKIN classification; both groups 20%) and changes in plasma NGAL and estimated GFR (*p* = 0.31 and *p* = 0.82, respectively). These findings are limited by the small sample size and use of the AKIN classification, but assume greater importance given the lack of randomised trial data for patients concerning renal outcomes.^86^ Similar findings have been reported in other small RCTs.^87^ However, potential findings of reduced inflammatory and procoagulant mediators is likely to further fuel investigation of MiECC.^88^

Further studies should assess renal outcomes, both immediate and delayed, using modern definitions, and comparing across varying techniques for CPB and with off-pump procedures. Considering the current literature, it should be noted that there is no standardised conventional system, which has implications for the interpretation of the above findings. There is increasing convergence of MiECC and more conventional systems with use of ‘optimised’ CPB systems, with reduced prime volumes, haemocompatible tubing and incorporated reservoirs. These changes can result in comparisons between systems, which appear to be artificially different and not reflective of systems in current use, and are therefore less applicable to contemporary clinical practice. Outcomes can be improved by clinicians incorporating the best practice elements which accompany much of the literature around MiECCs, with the aim of developing ‘optimised’ circuits. Similarly, recent European guidance has advocated consideration of elements of MiECC systems, including their incorporation into conventional systems.^89^

### Circuit priming

#### Retrograde autologous priming

RAP reduces haemodilution by priming the circuit with autologous blood, drained from the cannulation sites.^90^ A before-and-after study demonstrated no difference in AKI incidence (4.9% vs 4.8%, *p* = 0.95) (using the RIFLE classification), although UO on CPB was lower in the RAP group (510 mL vs 760 mL, *p* < 0.001).^90^ There were similar findings in a retrospective cohort study.^91^

One small RCT (*n* = 118) found no difference in AKI (a secondary endpoint) between RAP and non-RAP, but did reduce red blood cell transfusion, which is itself associated with CSA-AKI.^92^ A systematic review and meta-analysis identified six studies looking at AKI and found a similar incidence in RAP versus non-RAP (0.9% vs 0.4%; RR 1.63 [0.20–13.05]), although concluding this was of low certainty.^93^ A further meta-analysis has found similar results with regards to AKI, although once again red blood cell transfusion was reduced.^94^ European guidance recommends RAP (Class 1, Level A Evidence) on the basis of reduction in transfusion load, rather than a direct impact upon AKI.^89^

#### Priming fluid

The process of RAP will have an impact on priming fluid, by increasing the volume that is autologous blood and reducing the crystalloid/exogenous colloid volume. Other fluids have been investigated, and the use of mannitol has failed to reduce incidence of AKI in two RCTs.^95,96^ One small RCT found a non-significant reduction in a non-standardised definition of AKI using a circuit primed with 5% human albumin (and 0.9% saline) compared with 6% hydroxyethyl starch (HES) 130/0.4 (and 0.9% saline).^97^ In contrast, a retrospective study has reported an increase in AKI with HES prime.^98^ Another small (*n* = 84) RCT has examined a dextran-based priming fluid, in comparison with a crystalloid and mannitol solution, finding a reduction in a marker of renal tubular injury (N-acetyl-b-D-glucosaminidase (NAG)), but not in the incidence of AKI (18% vs 22%, *p* = 0.66).^99^

European guidance acknowledges the lack of consensus in optimal priming solution, although recommends against the use of starch solutions.^89^ In the absence of conclusive evidence, the use of balanced crystalloids with supplemental exogenous colloid, and RAP appears appropriate for renal outcomes.

### Pulsatile perfusion

Pulsatile flow has been described to increase mechanical energy transmission to the vessel wall, which induces vasodilator production, maintaining capillary patency and cellular perfusion.^100^ In contrast, non-physiological linear perfusion has been demonstrated to exacerbate organ injury, elevate peripheral vascular resistance, cause poor microcirculation, and increase tissue oedema.^101^ Emerging data from patients with continuous-flow ventricular assist devices may influence consideration of the importance of pulsatile perfusion.^9,100^

Two observational studies, both originating from the same single-centre, have provided evidence with regards to the impact of pulsatile perfusion. An initial, smaller study (*n* = 132) of matched patients undergoing pulsatile or non-pulsatile CPB demonstrated a non-significantly reduced requirement for RRT in the pulsatile group (4.5% vs 15%, *p* = 0.076). However, this was a small, non-randomised study, which excluded emergency operations.^102^ A subsequent larger (*n* = 2489), before-and-after study examined the difference following the introduction of pulsatile CPB at this centre. The authors found no difference in overall AKI incidence, or in the incidence of individual stages. Whilst this study was non-randomised and single-centre, it includes a good sample size, and used the KDIGO criteria for AKI (sCr alone).^103^ Therefore, the importance of pulsatile perfusion, and the way in which this is achieved, is ripe for future study.

Current European guidance advocates the consideration of pulsatile perfusion for the reduction of postoperative renal (and pulmonary) complications (Class IIa, Level B evidence), based on evidence from two meta-analyses.^89,104,105^ The first of these reported significantly greater creatinine clearance in the pulsatile perfusion patients compared with non-pulsatile perfusion, however postoperative creatinine was not significantly different between the two arms, and the studies included in the meta-analysis did not assign patients to each strategy based upon preoperative risk of renal dysfunction.^104^ The latter meta-analysis did report reduced incidence of acute renal insufficiency (corresponding to KDIGO Stage 1 AKI), but not acute renal failure (KDIGO Stage 3 – RRT requirement) with pulsatile perfusion.^105^ These findings support the call for further evidence.

### Perfusion targets

Common haemodynamic targets will include CPB flow rates equivalent to a cardiac index of 2.2–2.5 L/min/m^2^ with a mean arterial pressure (MAP) of 50–70 mmHg, aiming for maintenance within the range of organ autoregulation.^14^ Renal, and crucially medullary, oxygenation is important for avoidance of CSA-AKI, and oxygen delivery to the medulla is dependent upon several factors, including the CPB flow rates and MAP. Evidence from an animal model in sheep have suggested that oxygen delivery can be maintained by both strategies (i.e. increasing pump flow or increasing target MAP) in isolation. Alongside increasing medullary oxygenation, both strategies can increase creatinine clearance, albeit at supranormal values.^106^

#### Flow rates

As described in the preceding section, increasing flow rate may not increase organ blood flow, and maximal renal blood flow is markedly reduced. An influential RCT demonstrated increased CSA-AKI with renal oxygen delivery <272 mL/min/m^2^ (at temperature 32–34°C).^107^ More recent, but smaller, RCTs have demonstrated a favourable risk ratio (RR) for AKI-CPB with oxygen delivery >280 mL/min/m^2^ and >300 mL/min/m^2^, with RR 0.45 [0.25–0.83] for Stage 1 AKI and RR 0.49 [0.30–0.77] for all stage AKI.^108,109^ Furthermore, a goal-directed perfusion initiative which included targeted oxygen delivery >300 mL/min/m^2^ reduced incidence of AKI (23.9% vs 9.1%, *p* = 0.008), although this included several other interventions (MAP >70 mmHg and zero-balanced ultrafiltration).^110^

A large study of 19 410 patients used multivariate logistic regression modelling to identify and then validate the optimal DO_2_ for avoidance of AKI. Minimum DO_2_ was associated with any AKI (RIFLE classification) with an optimal threshold of 270 mL/min/m^2^. Furthermore, every 10 mL/min/m^2^ decrease in DO_2_ increased the likelihood by 7% for AKI, and there was an odds ratio (OR) 1.52 for AKI for those below the threshold.^111^

However, the use of higher flow rates needs to be considered in the context of the surgical requirements, often requiring low flow, for example during manipulation of the aorta, therefore surgical techniques are likely to supervene in this relationship. Again, further RCT evidence is required, although goal-directed therapy, to reduce postoperative complications, is recommended in the European guidance (Class I, Level A evidence).^89^

#### Mean arterial pressure targets

Maintenance of an elevated MAP does not have robust evidence supporting its adoption. Two moderately-sized RCTs (*n* = 300 and *n* = 197) with incidence of AKI as a primary and secondary outcome respectively, did not demonstrate an improved outcome with raised MAP (75–85 mmHg and 70–80 mmHg) compared with a lower MAP and unchanged flow rates (50–60 mmHg and 40–50 mmHg).^112,113^ Notably, in the latter study, the lower MAP target was markedly low, which may reduce wider applicability. Furthermore, whilst there was no difference in peak sCr (118.0 micromol/L (low MAP) vs 121.9 micromol/L (high MAP), *p* = 0.57), there was a greater incidence of a doubled sCr value (2.0% vs 9.4%, *p* = 0.03) in the higher MAP group.^113^ Whilst, this is a non-standardised AKI definition, it may underlie the fact that the attainment of a higher MAP will certainly involve the use of vasoactive agents, which will have their own deleterious effects on renal perfusion.^114^ A meta-analysis has supported the lack of impact upon incidence of AKI.^32^ Furthermore, this is in accordance with consensus European guidance, which advises against the use of vasopressors to maintain an artificially elevated (>80 mmHg) MAP (Class III, Level B evidence).^89^

There is no evidence to support maintenance of a higher MAPs, however, there is somewhat more robust evidence for the avoidance of a markedly low MAP taken from a large (*n* = 6523) single-centre retrospective study, where time-weighted MAP 55–64 mmHg and <55 mmHg was associated with increased odds for Stage 2/3 AKI and for RRT requirement.^115^ As such, whilst no upper limit of MAP should be targeted based upon current evidence, avoiding MAP <60–65 mmHg as much as surgery allows, and <50–55 mmHg as far as possible is appropriate. The finding that excursions of MAP below the lower limit of the cerebral autoregulation threshold are associated with AKI after cardiac surgery on CPB suggests that more bespoke MAP targets for the prevention of renal injury may be possible with adoption of cerebral oximetry index monitoring.^116^

Further evidence for bespoke MAP targets may be found from an observational study of 157 patients undergoing surgery on CPB in a single-centre. A change in MAP intraoperatively from preoperative baseline ≥ 26mmHg was independently associated with CSA-AKI.^117^ Similar findings were reported in a larger (*n* = 7247) retrospective study, where percentage change in systolic arterial pressure on CPB, compared with preoperative, was associated with CSA-AKI.^118^ Furthermore, an observational single-centre study reported that a cumulative duration of mean perfusion pressure (MAP – Central Venous Pressure) ≥ 20% below baseline was an independent predictor of CSA-AKI.^119^ Additional prospective data should be sought to further validate these findings.

### Other perfusion management

Numerous aspects of perfusion management have been indirectly linked to poorer renal outcomes via processes such as haemolysis, and have been reviewed elsewhere.^120^

#### Temperature management

One RCT and one multi-centre observational study have demonstrated an increased incidence of CSA-AKI with rewarming to, or above, 37°C.^121,122^ Based upon this, rewarming (and hyperthermic perfusion) may be more deleterious than cooling, as was previously thought.^67^ Whilst further RCT evidence for mild versus moderate hypothermia, and normothermia is required, there is also a need for further investigation on rewarming trajectories.

#### Biocompatible circuits

More physiologically compatible circuit coatings have been employed, including heparin-coating. These have reduced the incidence of a number of outcomes associated with renal dysfunction, but there has been little assessment of their direct impact on renal outcomes.^114,123^ They nonetheless should be considered to reduce general postoperative complications according to European guidance (Class IIa, Level B evidence).^89^

#### Conventional ultrafiltration

Conventional ultrafiltration (CUF) is theorised to reduce haemodilution and optimise fluid status by reduction of intravascular plasma water and removal of pro-inflammatory mediators.^124–126^ These potential beneficial effects need to be balanced against the necessity to ensure adequate intravascular volume and therefore renal perfusion.^127^

A meta-analysis of 12 studies (*n* = 8005) found no significant difference in the incidence of AKI between patients undergoing ultrafiltration and those that did not. A subgroup analysis compared different ultrafiltration strategies (CUF, modified ultrafiltration (MUF), zero-balanced ultrafiltration (ZBUF), or combination MUF/CUF) and showed no difference in AKI incidence.^128^ Furthermore, there was no difference in AKI incidence with removal of UF volume greater or less than 2900 mL (roughly equating to 40 mL/kg for a 70 kg adult). This contrasts with a single-centre retrospective study that found that weight-indexed CUF volume >32 mL/kg during CPB for elective cardiac surgery was associated with an increased incidence and severity of AKI.^124^ Other studies have failed to demonstrate a benefit for ultrafiltration in renal outcomes.^129^ These results indicate that conventional ultrafiltration during CPB may not be associated with improved renal outcomes.

## Conclusion

Renal dysfunction is one of the major complications of cardiac surgery and cardiac bypass. AKI has been the focus of much research, however, there are longer-term states of dysfunction, AKD and CKD, both of which are also associated with poorer outcomes in patients undergoing cardiac surgery. Whilst AKI remains the key to the dysfunction spectrum, AKD represents a longer period in which to intervene to improve outcomes.

CPB presents numerous physiological insults to the cardiac surgery patient, many of which act in a synergistic fashion, and are superimposed upon the other pre-existing and concurrent perioperative insults this unique patient group faces. The insults include haemodynamic instability, inflammation, neurohormonal activation, ischaemia-reperfusion injury, oxidative stress, and embolic phenomena.

Research to improve outcomes has involved tackling many of these pathophysiological features as avoidance of CPB is neither always possible nor desirable. Use of miniature extracorporeal circuits may be of continued future interest, and some elements of these circuits have made their way into standard practice. Retrograde autologous priming and the choice of priming fluid will also continue to generate further research interest.

The conduct of CPB is also important to improve outcomes. Whilst pulsatile perfusion may not be as important as previously thought, the available evidence suggests avoiding periods of lower MAP (<50–55 mmHg) and to consider higher flow rates when appropriate. Active rewarming is also associated with poorer outcomes, and a more passive approach is preferable.

Following these evidence-based points may serve to improve both short and long-term renal outcomes. However, further research is required into many facets of peri-bypass care, which should occur alongside investigation into the deleterious effects of CPB.
